# Utility of the point of care CD4 analyzer, PIMA, to enumerate CD4 counts in the field settings in India

**DOI:** 10.1186/1742-6405-9-26

**Published:** 2012-09-21

**Authors:** Madhuri Thakar, Bharati Mahajan, Nawaj Shaikh, Salman Bagwan, Suvarna Sane, Sandhya Kabra, Bharat Rewari, Mohamad Shaukat, Namita Singh, Peter Trevor, Ramesh Paranjape

**Affiliations:** 1 Department of Immunology, National AIDS Research Institute, G-73, MIDC, Bhosari, Pune, 411 026, India; 2 National AIDS Control Organization, New Delhi, India; 3 Clinton Health Access Initiative, New Delhi, India

**Keywords:** CD4+ T-cell count, HIV, Point-of-care, PIMA CD4 analyzer

## Abstract

**Background:**

In resource limited settings non-availability of CD4 count facility at the site could adversely affect the ART roll out programme. Point of care CD4 enumerating equipments can make the CD4 count available at the site of care and improve the patients’ management considerably. This study is aimed at determining the utility of a Point of Care PIMA CD4 analyzer (Alere, Germany) in the field settings in India.

**Method:**

The blood samples were collected from 1790 participants at 21 ART centers from different parts of the country and tested using PIMA and the reference methods (FACSCalibur, FACSCount and CyFlow SL3). The paired finger prick and venous blood samples from 175 participants were tested by the PIMA CD4 Analyzer and then by FACSCalibur.

**Result:**

The CD4 counts obtained by PIMA CD4 analyzer showed excellent correlation with the counts obtained by the reference methods; for venous blood the Pearson’s r was 0.921, p < 0.001 and the relative bias was 0.2% (range: -42 to 42%) and for finger prick samples, the Pearson’s r was 0.856 and the relative bias was −9.1% (range: -46% to 27%). For CD4 ranges; <250, 251–350, 351–500 and >500 cells/mm3, the differences in the median CD4 counts obtained by the reference method and the PIMA analyzer were not significant (P > 0.05) and the relative bias were low (−7 to 5.1%). The Intermachine comparison showed variation within the acceptable limit of%CV of 10%.

**Conclusion:**

In the field settings, the POC PIMA CD4 analyzer gave CD4 counts comparable to the reference methods for all CD4 ranges. The POC equipment could identify the patients eligible for ART in 91% cases. Adequate training is necessary for finger prick sample collection for optimum results. Decentralization of CD4 testing by making the CD4 counts available at primary health centers, especially in remote areas with minimum or no infrastructure would reduce the missed visits and improve adherence of the patients.

## Introduction

CD4 count estimation is a mainstay of monitoring the HIV disease progression and initiation and monitoring of anti-retroviral treatment (ART) [1-4]. The World Health Organization (WHO) has recommended using CD4 counts for initiation and monitoring of ART in HIV infected individuals in recourse limited settings [1]. The scaling up of the public ART programmes increased the demand for CD4 counts globally [5]. In a resource limited setting CD4 count facility is not available in peripheral areas and often the patients need to travel long distances or the samples need to be transported to the centres where the facility for CD4 count estimation is available. This could be a barrier for expansion and decentralization of the ART programme and adherence to treatment.

A point of care CD4 assay may be able to fill this gap and help in expanding the ART services and increase adherence. The Point-Of-Care (POC) CD4 equipments will be able to give the results in a very short time on the same day of collection and thus would avoid need for another visit for collecting results. PIMA CD4 analyzer (Alere) is one of the POC equipment. The closed disposable cartridge used in the analyzer contains the reagents required for the CD4+ T cell count estimation. It can also work on finger prick sample and give results within 20 minutes of the sample collection. The validation studies showed that the PIMA analyzer could give CD4 counts comparable to those obtained with the standard flow cytometers such as FACSCount and FACSCalibur [6-8]. For efficient working of the POC machines it is important to know the accuracy of the PIMA analyzer at different CD4 levels.

In the present study we assessed the feasibility of use of PIMA POC CD4 analyzer in the field setting in Indian ART programme. The PIMA CD4 analyzers were placed at 21 different ART centers in different parts of the country with maximum load of 25 patients per day. The Intermachine comparisons and repeatability of the CD4 counts were assessed using venous blood samples. The finger prick samples collected at the field settings were also tested for the accuracy of CD4 counts in comparison with the paired venous samples.

## Methods

### Study centres

The study was conducted at 21 ART centres having minimum (5-10/day) to moderate (25-30/day) patient load. Of these 21 centres, 2 had FACSCalibur, 13 had FACSCount (both from Becton Dickinson, USA) and 6 had CyFlow® SL3 counter from Partec, Germany. All these equipments are under the external proficiency assessment conducted with Quality Assessment and Standardization for Immunological measures relevant to HIV/AIDS, Public Health Agency, Canada (QASI) for the last three years and performing satisfactorily. Also the inter machine comparison between these equipments using fresh blood samples has been published earlier [9,10]. These equipments were considered as reference method in the study. The technologists from these centers were trained for two days for finger prick sample collection and the CD4 count estimation using PIMA analyzer including the use of calibrators. The equipments were handed over to the technologists during training.

### Study participants

During June to August 2011, each centre consecutively enrolled 5 to 10 HIV positive patients for CD4 count estimation every day after obtaining written informed consent for CD4 count estimation. Approximately 100 patients were enrolled by each centre. The inclusion criteria included age between 18 to 60 years and willingness to give blood sample for CD4 count estimation.

The paired finger prick and the venous blood samples were collected at NARI, Pune from 175 HIV infected individuals who gave written informed consent for collection of finger prick samples.

### Blood collection and processing

Three ml of venous blood sample was collected in K3 EDTA vacutainers. The capillary blood sample was collected from the finger tip using lancet finger stick. A puncture depth of 1.8 mm with a blade-type lancet (Sarstedt) was used to achieve sufficient capillary blood flow. The venous or the finger prick samples were first tested in the ART centre using PIMA CD4 analyzer and the results were stored in the centre. The samples were then sent to the attached laboratory for processing by the respective reference methodologies. The technicians estimating CD4 count by reference method were blinded for PIMA results. The CD4 counts obtained by the reference method were provided to the patients.

### CD4 count estimation

#### Test method- pima CD4

The samples were run in the PIMA CD4 analyzer only after the normal and Low value control cartridges gave acceptable value. After collection of venous sample, 25 μl of blood was immediately added to the PIMA CD4 cartridge. In case of finger prick sample, the sample was directly collected on the cartridge. The cartridge was capped and inserted immediately into the PIMA analyzer. The PIMA analyzer works on volumetric principle. Hence every time 5 μl of blood was drawn into the detection channel of the PIMA cartridge from the blood added in the receptacle.

During the incubation, freeze-dried fluorescently labeled antibodies (anti-CD3 and anti-CD4) get mixed with the blood and the images of the CD3+ and CD4+ cells are captured by the camera and the results are then expressed as cells/μl within 20 minutes. In the study, the print out of the CD4 count obtained from the PIMA analyzer was checked by the supervisor and stored in the clinic itself.

### Reference method-flow cytometry

In the laboratory, the blood samples were tested by respective reference methodology on the day of collection. The daily calibration and internal quality controls were included in every run. The samples were acquired and analyzed only when the quality control indicators were passed.

#### FACSCalibur (Becton Dickinson, USA)

Twenty μL of liquid antibody reagent (MultiTEST CD3 FITC, CD4 PE, CD45 PerCP, Becton Dickinson, USA) and 50 μL of whole blood was added to the TruCOUNT tube (Becton Dickinson, USA) containing the reference beads. After incubation, the RBCs were lysed using 450 μl of 1:10 diluted lysing solution for 15 minutes in the dark at ambient temperature. The stained sample was acquired on FACSCalibur (Becton Dickinson, USA) and analyzed automatically using the MultiSET software (BD Biosciences) by gating CD45+ T cells in first place and these cells were further gated in CD3 + CD4+ versus CD3 + CD4- T cells.

#### FACSCount (Becton Dickinson, USA)

Fifty μL of whole blood was added to FACSCount tube containing CD3 PE /CD4 PE.Cychrome (PE.Cy5) monoclonal antibodies and a known number of reference beads in a liquid format (Becton Dickinson, USA). After incubation, 50 μL of fixative (5% formaldehyde in PBS) was added. The stained sample was acquired and analyzed on the FACSCount using the automated analyzing software by gating on CD3 + CD4+ T cells.

#### CyFlow SL3 (partec, Germany)

Twenty μL of whole blood and 20 μL of CD4-PE (Partec, Germany) monoclonal antibody were added to the sample tube (Partec, Germany). After incubation, 800 μl of no lyse buffer (Partec, Germany) was added into the sample tube. The stained sample was then acquired on the CyFlow SL_3. The acquired data was analyzed using the inbuilt CyView software by gating on the histogram of CD4+ T cells. The histogram and absolute counts are displayed and printed automatically. The results were printed and stored after review.

### Assessment of inter machine variation for PIMA CD4 analyzer

Before distribution of the machines to respective centers, the inter-machine variation was assessed by running the commercially available stabilized blood samples with Normal and low level CD4 count (Immuno Trol controls, Beckman Coulter, USA) and 2 freshly collected blood samples with low and high CD4 counts.

Additionally the onsite inter machine variation was assessed using the low (CD4 count range: 129 to 201 cells/mm^3^) and normal (CD4 count range: 857 to 1075 cells/mm^3^) level PIMA Bead standards which were used by these centers daily.

### Statistical analysis

For assessing the precision as well as inter-machine variability amongst all 21 machines, the percent CV value less than 10% was considered to be an acceptable value.

The Pearson’s correlation coefficient was used to estimate the strength of the correlation between the CD4 counts obtained by the PIMA CD4 analyzer and the respective reference methods. The data was analyzed using Bland-Altman analysis to find out whether the methods agree sufficiently well [11]. Since the data was collected on a wide range of CD4 counts the relative bias was calculated which is expected to normalize wide range of absolute count data and thus would allow direct comparison between PIMA and various reference methods. The relative bias was calculated by converting the ratio of the difference between the CD4 counts obtained from two machines and the average of both readings into percentages.

Similar analyses were performed for comparison of the finger prick sample with the venous sample. To determine the accuracy of the PIMA analyzer at different CD4 levels, the median values obtained for different CD4 ranges (<250, 251–350, 351–500 and >500 cells/mm^3^) by both, the reference method and the PIMA analyzer were compared. The relative bias was also calculated for these CD4 ranges. To determine the clinical significance of the variations in the CD4 estimations in the decision on treatment initiation, the sensitivity and specificity of PIMA CD4 analyzer to identify patients requiring ART (CD4 count < 350 cells/μL) was determined.

## Results

Before distribution, the inter machine variation for all 21 machines was assessed using fresh as well as stabilized blood samples with high and low CD4 counts. Using the stabilized blood samples, the% CV was found to be 4% and 3% for low (130 cells/mm^3^) and high (649 cells/mm^3^) Immuno Trol controls respectively. Whereas for the freshly collected samples, the%CV was 10% for low count (180 cells/ mm^3^) and 8% for the normal count (749 cells/mm^3^). When the machines were installed on site, the onsite variation was assessed by the values of low and normal level PIMA Bead standards which were used by all centers daily before processing the samples. The mean% CV of all the centers for low control was 2.3 (range 0.67 -7.59) where as the mean%CV for high control was 1.6 (range: 0.39- 7.19). The data collected for the inter machine comparison between the three reference analyzers at a single center at two different out-patient clinics showed that the% CV between these analyzers was 8% with a range of 2.4% to 9% (N = 25, range of CD4 count: 114–965).

**Figure 1:**
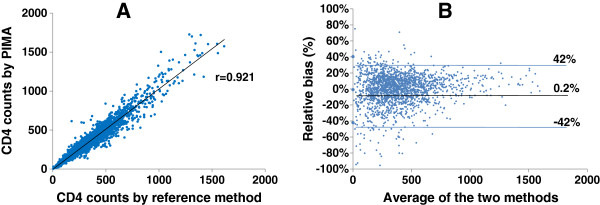
**Comparison of the CD4 counts obtained by PIMA analyzer and reference methods using venous blood from 1790 samples.** Figure 1A: Linear regression analysis: The CD4 counts obtained by PIMA CD4 analyzer at 21 centers are plotted on Y axis and the counts obtained by the respective reference methods are plotted on X axis. Figure 1B: The agreement analysis using the Bland Altman plots. The percent relative bias for PIMA analyzer and the respective reference method is plotted on the Y axis and the average of the CD4 count by both the methods is plotted on X axis. The black line represents the mean% relative bias where as the blue lines represent the range of Mean ± SD.

A total of 1790 participants were enrolled in the study (958 Male and 832 Female). The median age of the participants was 36 years (range: 19 to 55 years). The data showed excellent correlation between the CD4 counts obtained by PIMA CD4 analyzer and the respective reference methods (N = 1790, r = 0.921, p < 0.001, Figure 1A) at all centers. The Bland Altman plot analysis for the agreement between the values obtained with PIMA CD4 analyzer and the respective reference methods showed low relative bias of 0.2% (Mean ± SD: -42 to 42) (Figure 1B). The CD4 counts obtained using individual reference methods also showed good correlation with the counts obtained using PIMA CD4 analyzer FACSCount (r = 0.983, P < 0.01), FACSCalibur (r = 0.988, P < 0.01) and Cyflow SL3 (r = 0.977, P < 0.01). The CD4 counts obtained using PIMA analyzer were compared with those obtained using either of the three reference methods at different clinically relevant CD4 levels such as <250, 251–350, 351–500 and >500 cells/mm^3^ (Table 1). The differences in the median CD4 counts obtained by the reference method and the PIMA analyzer were not significant (P > 0.05) for all CD4 ranges. The relative bias was also ranged between −7 to 5.1%.

**Table 1:** Comparison of CD4 counts obtained by PIMA analyzer and the reference methods within different CD4 ranges

**CD4 Range (Cells/mm**^**3**^**)**	**N**	Median CD4 Count (Range)* Cells/mm^**3**^		**Mean relative bias (±2SD)**
		**Reference Method**	**PIMA Analyzer**	
All	1790	356 (2–1726)	354 (6–1615)	−1% (−43, 42)
<250	508	165 (2–250)	168 (6–348)	−7% (−65, 51)
<350	877	228 (2–350)	225(6–542)	5% (−45,55)
251-350	369	298 (251–350)	299 (153–542)	−2% (−40, 36)
351-500	423	417 (351–500)	415 (213–676)	0% (−30, 30)
>500	490	648 (502–1726)	637 (179–1615)	5.1% (−23, 33)

*: The differences between the median CD4 counts obtained by the one of the reference methods and the PIMA analyzer was statistically insignificant for all CD4 ranges (P > 0.05).

WHO recommends to initiate ART at CD4 count < 350 cells/mm^3^(1). Hence, the sensitivity and specificity of the PIMA CD4 analyzer to identify patients with CD4 count <350 cells/mm^3^ was found to be 91% indicating that the chances of 9% wrong counts. When the data within the CD4 range of 251–500 cells/mm3 was assessed it was observed that 50% of the mismatched values were within the 320 to 380 cells/mm^3^. The data was analyzed for individual reference analyzer (Table 2) which also showed the sensitivity and specificity of the PIMA analyzer ranged between 91 to 96% and the mean relative bias between −5 to 8%.

**Table 2:** Sensitivity of PIMA analyzer to identify patients with CD4 count <350; a cut off used for ART initiation

**Reference method**	**PIMA analyzer**			
	**N**	**Sensitivity**	**Specificity**	**Mean Relative bias (±2SD)**
FACSCalibur	121	96%	91%	4(−48, 56)
FACSCount	206	92%	91%	−5( −49, 59)
Partec	550	91%	96%	8(−8.4, -7.6)

**Figure 2:**
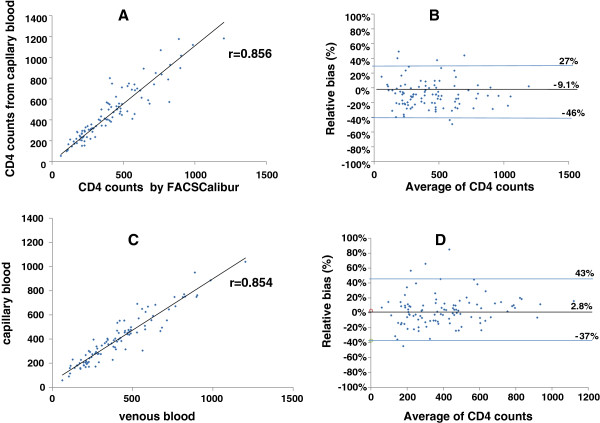
**Comparison of the CD4 counts obtained from finger prick sample and from paired venous blood.** Figure 2A and C: Linear regression analysis: The correlation between the CD4 counts obtained from finger prick sample using PIMA CD4 analyzer (Y axis) and from venous blood using FACSCalibur (2A) and PIMA (2C) (X axis) is assessed. Figure 2B and D: The agreement analysis using the Bland Altman plots. The percent relative bias for CD4 counts from finger prick sample and the paired venous blood using FACSCalibur (Figure 2B) and PIMA (Figure 2D) is plotted on the Y axis and the average of the CD4 count by both the samples is plotted on X axis. The black line represents the mean% relative bias of where as the blue lines represent the range of Mean ± SD.

In 175 patients, the finger prick sample was obtained in addition to venous blood. The CD4 counts obtained from the finger prick samples using PIMA CD4 analyzer correlated well with the CD4 counts obtained by reference method, FACSCalibur using the venous blood (r = 0.856, P < 0.01, Figure 2A) with a relative bias of −9.1%(range: -46% to 27%, Figure 2B). The CD4 counts obtained using PIMA analyzer on both, finger prick and venous blood were also comparable with r = 0.854 and slightly better relative bias on 2.8% (range: -37% to 43%) (Figure 2C and D).

The users of the equipment at various centers expressed that the analyzer was compact and hence could fit in the small space available at the centers. The equipment is battery operated, showed a battery backup of 3–4 hours eliminating the requirement of continuous electricity. The rate of invalid cartridges was 1 to 2 percent at these centers and thus repeat testing needed to be done in such cases.

## Discussion

The present field based study showed that the PIMA analyzer gave comparable results with the reference methodologies for different CD4 ranges; one of the important characteristics for the validation of a new technology. The study could analyze more than 300 samples (range 369 to 508) samples at each CD4 range; <250, 251–350, 351–500 and >500 cells/mm^3^ and the analysis showed very low relative bias within the PIMA and reference methodologies. Before distribution of the machines to various centers the inter machine comparison showed less than 10 percent%CV for both high and low CD4 counts for either the stabilized blood samples or fresh blood samples indicating good precision. The onsite inter-instrument variation was also within the 10% limit indicating the efficient use of PIMA analyzer as POC machine. The sensitivity and specificity of the PIMA analyzer to identify the patients with CD4 count less than 350, was found to be 91%. This indicated that the use of PIMA would put additional burden on the treatment providers as the treatment would be given to the patients who still have CD4 counts above 350. However the analysis showed that more than 50% mismatched results were between CD4 counts of 320 to 380; indicating that most of the patients would need ART in short time. The previously reported study has showed higher sensitivity but lower specificity to identify patients with CD4 counts lower than 350 cells (8). Previous studies have also shown less precision for the finger prick samples and also indicated that the finger prick sample collection required adequate training for correct use of the samples (7,8,12). Our study confirmed that the finger prick samples could be tested on PIMA analyzer with slightly less efficiency and also confirmed the requirement of the operator training for proper collection of finger prick sample to avoid multiple pricks. It was observed that in case of insufficient and improper finger-prick sample collection, the reliability of the CD4 counts by PIMA analyzer was questionable. Also our study participants preferred to give venous blood sample. The primary reasons were requirement of blood collection for other investigations using venous blood and a fear of being subjected to multiple pricks if sufficient volume of blood is not obtained in a single prick. Hence the finger prick blood sample could be the method of choice when only CD4 testing has to be carried out. The PIMA CD4 analyzer is battery operated and the reagents are stable at room temperature making it suitable for use in the areas where the reliable electric supply is not available and the ambient temperatures are often high. The cartridges have shelf life of six months making it suitable for remote areas where the shipment, delivery and storage, would be difficult. The testing does not require any additional equipment. The analyzer can be used after a minimum training and can be run by the clinical staff such as nurses where multi-tasking may be necessary due to shortage of trained staff or lack of sufficient workload [12].

The stabilized blood samples could be tested reliably by PIMA analyzer indicating amenability of the system for participation in the External Quality Assurance Scheme.

Unlike other CD4 enumerating equipments, in PIMA analyzer, the complete process of staining takes place in the equipment itself. Hence the sample throughput would be relatively low and the analyzer would be useful at the centres with 10–20 patients load/day. However, it is not expected that peripheral centres will have higher patient load and more than one machine could be installed in the centres if required.

The countrywide national ART roll out programme in India comprises of 300 ART centers and 550 linked centers with 3,64,000 adult populations on ART by January 2011 [13].The availability of CD4 counts in selected centers required either long distance travel for the patient or transport of the sample to the laboratory. This resulted in missed visits of the patients and problems in patient management. The loss to follow up of HIV infected patients after the diagnosis has been reported to be in the range of 16 to 25% in India [14], personal communication]. Although multiple factors are responsible for loss to follow up, availability of CD4 testing at point of care will help in reducing the number of clinic visits, thus helping in reducing loss to follow up. A recent report from Africa showed that POC CD4 testing could successfully reduce the pretreatment loss to follow-up [15]. Also the implementation of 350 CD4 count as a cut off for ART initiation instead of earlier cutoff of 250 would increase the burden on CD4 testing facility. Hence the decentralization of CD4 testing by providing CD4 count at primary health centers in combination with HIV diagnosis could enable proper monitoring of disease progression and ART initiation at the primary health centers only and would maximize the public health benefit of POC technology.

The PIMA analyzer does not provide percentage of CD4+ cells; hence, it would not be useful for monitoring the paediatric population younger than 5 years. As the movement of the paediatric patients/samples is difficult in the periphery, unavailability of CD4 percentage at the peripheral centre could be one of the limitations of this POC machine. In such cases the dual platform technology can be used by using absolute lymphocyte counts from hematology analyzer or from peripheral blood smear and the absolute CD4 counts from PIMA analyzer to calculate CD4 percentages. However the disadvantages would be of influence of the variation due to another system.

The cost of the PIMA analyzer is lesser than the available single platform CD4 machines and the cost of the cartridge is around 10 US$ which is slightly higher which might pose problem if not procured in bulk amount where the cost benefit could be obtained.

In conclusion, the study showed that the POC PIMA CD4 analyzer would be suitable for use in remote areas with minimum or no infrastructure. The availability of the CD4 counts on the same day of sample collection would reduce the number of repeat visits and improve patient management. Hence the integration of POC CD4 testing into the national AIDS control program would facilitate the better patient management in HIV infection.

## Competing interests

The authors have declared that no competing interests exist.

## Authors’ contributions

MT: Planning, execution, analysis of the data and manuscript writing. BM, NS, SB: Execution of the study, data collection and training. SS: statistical analysis. SK, BR, MS, NS, PT: Execution and manuscript writing. RP: Planning and manuscript writing. All authors read and approved the final manuscript.
